# Comparative transcriptomics of the nematode gut identifies global shifts in feeding mode and pathogen susceptibility

**DOI:** 10.1186/s13104-016-1886-9

**Published:** 2016-03-05

**Authors:** James W. Lightfoot, Veeren M. Chauhan, Jonathan W. Aylott, Christian Rödelsperger

**Affiliations:** Department for Evolutionary Biology, Max-Planck Institute for Developmental Biology, Spemannstr. 35-39, Tübingen, Germany; Laboratory of Biophysics and Surface Analysis, School of Pharmacy, University of Nottingham, Boots Science Building, Nottingham, UK

**Keywords:** Gene expression, Intestine, Hedgehog signaling, Immunity

## Abstract

**Background:**

The nematode *Pristionchus pacificus* has been established as a model for comparative studies using the well known *Caenorhabditis elegans* as a reference. Despite their relatedness, previous studies have revealed highly divergent development and a number of morphological differences including the lack of a pharyngal structure, the grinder, used to physically lyse the ingested bacteria in *C. elegans*.

**Results:**

To complement current knowledge about developmental and ecological differences with a better understanding of their feeding modes, we have sequenced the intestinal transcriptomes of both nematodes. In total, we found 464 intestine-enriched genes in *P. pacificus* and 724 in *C. elegans*, of which the majority (66 %) has been identified by previous studies. Interestingly, only 15 genes could be identified with shared intestinal enrichment in both species, of which three genes are Hedgehog signaling molecules supporting a highly conserved role of this pathway for intestinal development across all metazoa. At the level of gene families, we find similar divergent trends with only five families displaying significant intestinal enrichment in both species. We compared our data with transcriptomic responses to various pathogens. Strikingly, *C. elegans* intestine-enriched genes showed highly significant overlaps with pathogen response genes whereas this was not the case for *P. pacificus*, indicating shifts in pathogen susceptibility that might be explained by altered feeding modes.

**Conclusions:**

Our study reveals first insights into the evolution of feeding systems and the associated changes in intestinal gene expression that might have facilitated nematodes of the *P. pacificus* lineage to colonize new environments. These findings deepen our understanding about how morphological and genomic diversity is created during the course of evolution.

**Electronic supplementary material:**

The online version of this article (doi:10.1186/s13104-016-1886-9) contains supplementary material, which is available to authorized users.

## Background

Superficially, nematodes can be regarded as rather simple animals. They have a simple body plan, which in case of *Caenorhabditis elegans* is the outcome of a completely deterministic developmental proccess resulting in a fixed number of cells and large parts of their bodies are composed of two organs serving digestion and reproduction. However, the fact that nematodes form one of the most successful animal phyla and individual nematode species have invaded almost all ecological niches suggest that their relatively simple developmental program harbors enormous potential for adaptation to complex environments. This includes multiple independent events leading to the evolution of parasites that adapted to a diverse range of host environments (see [1, 2] for review). To understand, how such immense phenotypic and genotypic diversity is generated, is one of the key questions in evolutionary biology.

Over the last two decades, the nematode *Pristionchus pacificus* has been established as a satellite model organism to the widely known *C. elegans* for comparative studies involving developmental biology [3, 4], neuroscience [5, 6], immunity [7, 8], as well as comparative and population genomics [9, 10]. Despite the fact that both *C. elegans* and *P. pacificus* belong to the same taxonomic subgroup, Rhabditina, within nematodes [11], work on *P. pacificus* has revealed highly divergent patterns even involving newly acquired phenotypic traits [4] as well as novel genes [12]. One of the most striking examples of a novel trait in *P. pacificus* is the presence of a mouthform plasticity in *Pristionchus* nematodes. This describes an environmentally controlled irreversible decision to develop either one mouthform that is better suited for bacterial feeding or another mouthform that allows predation on other nematodes [4]. A second important morphological difference between *C. elegans* and *P. pacificus* nematodes, is the absence of a pharyngal structure in the terminal bulb of *P. pacificus*. The so called grinder is used to physically lyse bacteria in *C. elegans*. Therefore, typically, intact bacteria are not found in the gut of *C. elegans*, however, mutants defective in grinder formation exhibit intact bacteria in the gut [13]. It has been shown that the grinder was lost in diplogastrid nematodes to which *P. pacificus* belongs [14] and it also has been suggested that the absence of the grinder has important consequences on the susceptibility to certain pathogens [7] potentially leading to a greater resistance in *P. pacificus*. Obviously, these rather dramatic morphological differences between *P. pacificus* and *C. elegans* likely reflect different lifestyles and environments. While *Pristionchus* nematodes are found in a necromenic association with scarab beetles [15], so far, the ecology of *C. elegans* is only recently beginning to be understood [16, 17]. However, based on population genetic analysis, a recent bottle neck and strong selective sweeps in the last centuries suggested that the dispersal of *C. elegans* might be linked to human migration patterns [18].

To complement current knowledge about developmental and ecological differences between both nematodes with a better understanding of the differences in feeding modes, we have sequenced the intestinal transcriptomes of *C. elegans* and *P. pacificus*. Using previously published data sets of intestine-enriched genes to assess the quality of our *C. elegans* intestinal transcriptome, we use the *P. pacificus* intestinal transcriptome to ask, to what extent are the intestinal transcriptomes conserved and whether transcriptomic differences have implications on the intestinal environment and on susceptibility to certain pathogens.

## Methods

### Dissection of nematode intestines and RNA extraction

Young adult *C. elegans* (N2) and *P. pacificus* (PS312) nematodes were selected from NGM plates seeded with *Escherichia coli* (OP50). Animals were picked into 20 $\upmu$l M9 on a glass slide and carefully decapitated using a fine needle. Intestines were gently extracted and cut from the carcass which was subsequently disposed of while the intestines were suspended separately in 50 $\upmu$l of M9 in an Eppendorf tube. In total 250 intestines from each species were collected and processed for RNA extraction. The intestinal RNA was purified using an Invitrogen PureLink RNA Micro Kit (Catalog no. 12183-016) with slight modifications. Briefly, the intestines were incubated for 5 minutes with 250 $\upmu$l TRIzol at room temperature before the addition of 70 $\upmu$l chloroform and a further 2–3 min incubation. The samples were then centrifuged at 13,000 rpm at 40 $^\circ$C for 15 min and the upper phase containing the RNA transferred to a new tube and an equal volume of 100 % ethanol added. The binding, wash and elution steps were performed as described in the manufacturers manual.

### Transcriptome sequencing and analysis

RNA-seq libraries were generated using the Illumina TruSeq protocol and were sequenced as 100 bp paired ends in one multiplexed lane of an Illumina HiSeq 2000 resulting in 38,836,876 reads for the *C. elegans* intestine, 49,743,412 reads for the *C. elegans* whole animals, 47,369,694 reads for the *P. pacificus* intestine, and 42,912707 reads for the *P. pacificus* whole worms. Raw reads have been submitted to the NCBI short read archive under the study accessions: SRP061927 and SRP061928. We mapped raw reads to the *C. elegans* (WS230) and *P. pacificus* (Hybrid1) genome assemblies using tophat (version v2.0.3) and ran Cuffdiff (version v2.0.1) for differential gene expression analysis using the *C. elegans* (version WS230) gene annotations and the *P. pacificus* (version TAU) gene annotations. Protein domain annotations as well as orthology assignments were taken from [9, 19].

### Imaging *P. pacificus* luminal pH with extended dynamic range pH sensitive nanosensors

Extended dynamic range pH-sensitive nanosensors were produced and calibrated as reported previously [20]. Young adult *P. pacificus* nematodes were selected from synchronized NGM plates for intestinal pH measurements and immobilised on agarose pads. However, unlike in *C. elegans*, nanosensors were not maintained in the intestinal lumen of *P. pacificus* via feeding, therefore extended dynamic range pH-sensitive nanosensors (30 mg/ml) were introduced into the lumen of the *P. pacificus* intestine via microinjection. After successful injection, nematodes were placed onto OP50 seeded NGM plates and allowed to recover for 10 min before again being immobilised on fresh agarose pads for imaging. Green and red fluorescent channels were acquired on an Olympus FV 1000 confocal microscope and subsequently images were processed using MATLAB and FIJI open source software as previously described [20]. The pixel-wise ratio of green and red fluorescent channels facilitated accurate real-time pH analyses which were subsequently displayed as a false colour pH heat map.

## Results

### Identification of intestine-enriched genes in *C. elegans* and *P. pacificus*

**Fig. 1:**
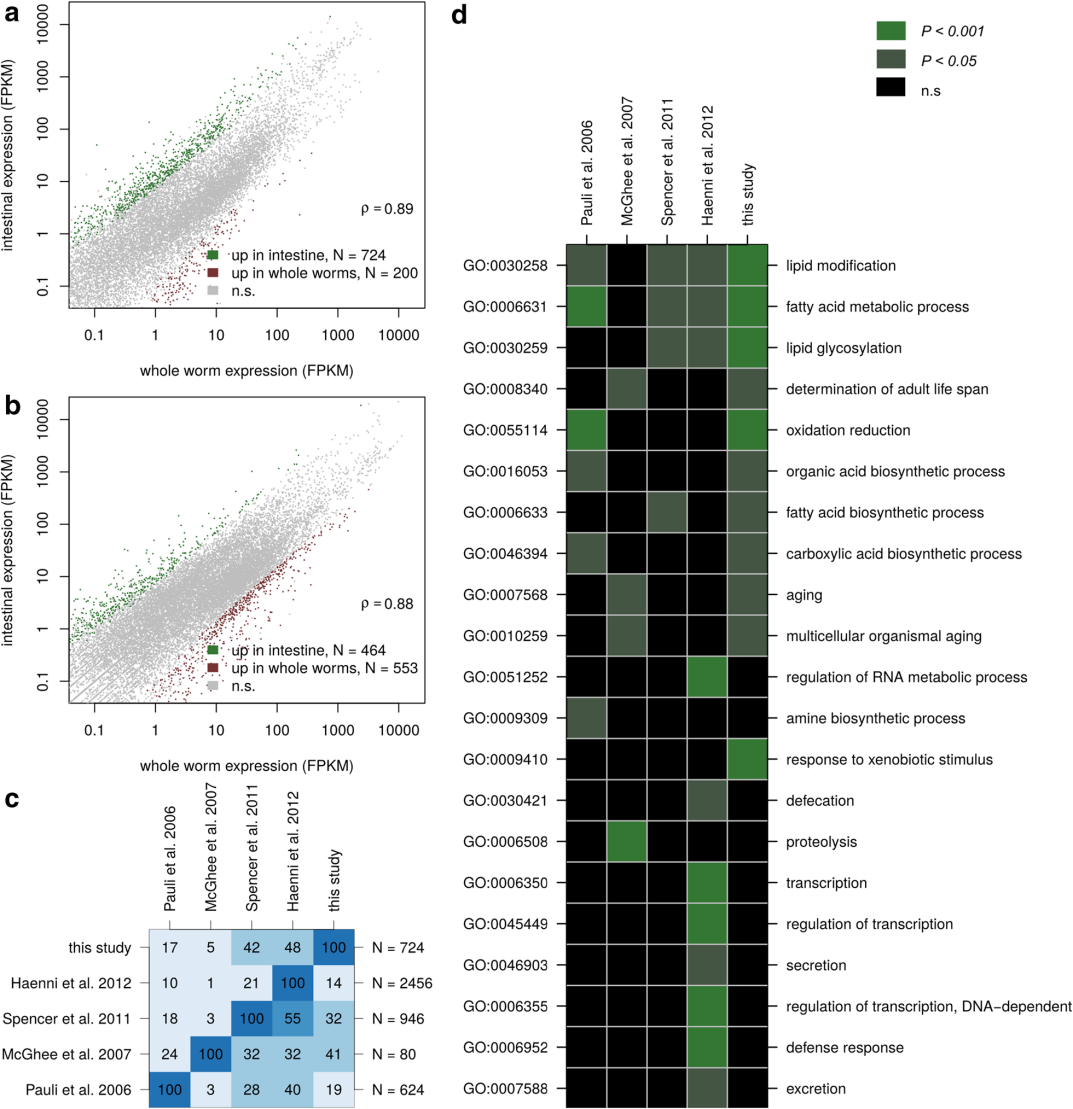
Sequencing of intestinal transcriptomes and comparison within *C. elegans*. **a** Correlation between estimated expression values in the intestinal transcriptome as well as the whole worm transcriptome in *C. elegans*. **b** Correlation between estimated expression values in the intestinal transcriptome as well as the whole worm transcriptome in *P. pacificus*. **c** Percentage of shared genes across five different *C. elegans* studies. For identified gene set (rows), we tested what percentage of genes was also found to be intestine-enriched for all other studies (columns), e.g. 42 % of the 724 genes identified in our study were also identified by Spencer et al. [25]. **d** Agreement in enriched Gene Ontology terms across different *C. elegans* studies

We dissected the intestines of *C. elegans* and *P. pacificus* adult animals and generated RNA-seq libraries that were sequenced in parallel with libraries of adult animals of both species. For both species, gene expression levels obtained from dissected as well as whole worms were highly correlated (Fig. 1a, b), suggesting that the relative normalization of expression levels relative to the expression of all genes, as commonly applied in analysis of RNA-seq data [21, 22], is indeed valid and that therefore both data sets are comparable.

Differential expression analysis identified 724 *C. elegans* and 464 *P. pacificus* genes that are enriched in the intestine as compared to whole animals (Additional file 1). The intestinal transcriptome of *C. elegans* has already been subject to several studies [23–26]. Pauli et al. immunopreciptated a poly-A tail binding protein that was transcribed from an intestine-specific promoter and identified 624 intestine enriched genes by comparison against muscle and germline specific expression [23]. McGhee et al. hand-dissected the intestines from two thousand gonad-less *C. elegans* glp-4(bn2) animals and identified 80 intestine-enriched genes by comparison against transcriptome data obtained from intact adults [24]. Spencer et al. used a cell sorting approach to isolate GFP labeled cells of various tissues followed by gene expression profiling on tiling arrays. Based on a comparison to whole worm expression data, they identified 924 intestine-enriched genes from late *C. elegans* embryos [25]. Haenni et al. employed a protocol to isolate GFP-labeled intestinal nuclei and compared the transcriptome of intestinal nuclei to a transcriptome from unsorted nuclei, resulting in a candidate gene set of 2456 intestinal-enriched genes [26]. We compared our set of 724 intestine-enriched genes with all four previous *C. elegans* intestinal transcriptome profiling studies (Fig. 1c). Despite drastic differences in previous approaches (various protocols to enrich for intestinal transcripts, different developmental stages, usage of mutant lines), which might explain to a large extent discrepancies in the shared gene sets (Fig. 1c), 477 (66 %) of our *C. elegans* intestine-enriched genes were identified previously by at least one other study. Thus, our data set is in good agreement with previous studies of *C.elegans* intestinal transcriptomes [23–26]. To investigate similarities across different gene sets at a different level, we repeated gene ontology (GO) enrichment analysis for all five different *C. elegans* data sets. Interestingly, not a single GO term was significantly enriched in all five data sets (Fig. 1d). The most robustly identified GO terms are all related to fatty acid metabolism. All of these most frequently found GO terms were also found based on our data, again supporting that our study is to a large extent in agreement with common trends identified by previous studies [23, 25, 26].

### Highly diverged intestinal transcriptomes between *C. elegans* and *P. pacificus*

**Fig. 2:**
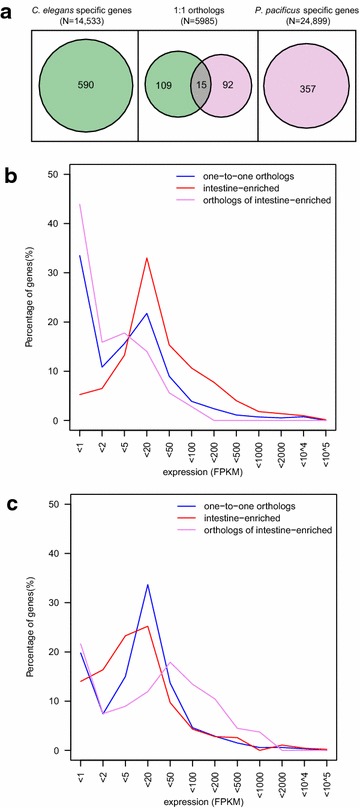
Intestinal transcriptomes are highly diverged. **a** Number of lineage-specific genes in the *C. elegans* and *P. pacificus* intestinal transcriptome and *Venn Diagram* showing intestine-enriched genes with one-to-one orthology relationship across both species. Only 15 genes were found that have such a one-to-one correspondance and that were identified as intestine-enriched in both species. **b** Expression levels of various gene sets in *C. elegans*. **c** Expression levels of various gene sets in *P. pacificus*

To assess the degree of conservation between the *C. elegans* and *P. pacificus* intestinal transcriptomes, we first focused on the comparison of one-to-one ortholougous gene pairs. For the set of 5985 predicted one-to-one orthologs [9, 19], the numbers of intestine-enriched genes condensed to 124 for *C. elegans* and and 107 for *P. pacificus* (Fig. 2a). This indicates that the vast majority of intestine-enriched genes is constituted of lineage-specific genes that either result from duplication or gene losses in at least one of the lineages or that have unknown origin, because no homologs in the other lineage could be identified. A strong signature of lineage-specific genes was already observed among genes showing developmental regulation in *P. pacificus* [19], indicating that spatial and temporal gene expression patterns are strongly impacted by duplication events. For one-to-one orthologous genes, that were most likely present as a single copy in the ancestor of *C. elegans* and *P. pacificus*, only 15 cases ($P<10^{-8}$, Fisher’s exact test) could be found where the *C. elegans* and the *P. pacificus* copy were identified as significantly upregulated in the intestinal sample as opposed to the whole worm. This suggests a large degree of functional divergence that reflects findings from other comparative studies between *C. elegans* and *P. pacificus* [3, 27] and has also been observed at much shorter evolutionary periods [28]. In order to test whether conservation of tissue-specific expression cannot be detected, because an intestine-enriched transcript has acquired broad expression in either of the species, we investigated the distribution of intestinal expression values for intestine-enriched genes, orthologs of intestine-enriched genes in the other species, and compared these to the expression of all genes with one-to-one orthologs. As expected, compared to genes with one-to-one orthologs, intestine-enriched genes are shifted towards higher expression values, i.e. show a significant depletion ($P <0.01$, Fisher’s exact test) of lowly expressed genes (FPKM $< 1$, Fig. 2b, c). However, 44 % of intestine-enriched genes in *P. pacificus* and 22 % of intestine-enriched genes in *C. elegans* show very low expression (FPKM $< 1$) in the intestinal transcriptome of the other species, indicating that a large fraction intestine-enriched genes is species-specific. However, a subset of intestine-enriched genes in *C. elegans* also show unusually high expression in *P. pacificus* (Fig. 2c) suggesting that at least to some extent the lack of conservation can be explained by broad expression of intestine-enriched genes in one of the lineages.

The fifteen intestinal genes with conserved intestinal expression are shown in Table 1. Interestingly, there are three Hedgehog signaling genes in this list. Hedgehog signaling has been shown to have important roles in development of intestines of vertebrates [29] and flies [30], and also in *C. elegans*, it has been shown that RNAi inhibition of ptr genes caused an abnormal accumulation of fluid-filled vacuoles in the intestines [31]. Thus our analysis further supports a highly conserved role of Hedgehog signaling in animal intestinal development.

**Table 1:** *C. elegans* genes with *P. pacificus* one-to-one ortholog, which showed intestine -enriched expression in both nematodes

Sequence ID	Gene symbol	*P. pacificus* ortholog	Description
Y65B4BR.6	grl-16	Contig60-snapTAU.52	Hedgehog-like protein
C45B2.7	ptr-4	Contig56-snapTAU.40	Hedgehog receptor protein
F46G10.5	ptr-24	Contig85-snapTAU.55	Hedgehog receptor protein
W04G3.8	lpr-3	Contig50-snapTAU.173	Lipid transporter
W04G3.2	lpr-5	Contig50-snapTAU.171	Lipid transporter
T14B4.6	dpy-2	Contig5-snapTAU.522	Collagen
F46C8.6	dpy-7	Contig56-snapTAU.167	Collagen
Y69A2AR.4	smf-3	Contig11-snapTAU.428	Metal ion transporters
W07G1.3	zip-3	Contig41-snapTAU.107	bZip transcription factor
C50B6.7	NA	Contig43-snapTAU.73	Amylase
Y71H2AM.13	NA	Contig11-snapTAU.568	Carboxylesterase
H04M03.4	glf-1	Contig109-snapTAU.67	UDP-galactopyranose mutase
F30H5.3	NA	Contig11-snapTAU.188	Peptidase inhibitor
F31D4.5	NA	Contig20-snapTAU.138	Unknown
ZK682.5	lron-2	Contig41-snapTAU.192	Unknown

### Intestinal transcriptomes are dominated by different gene families

**Fig. 3:**
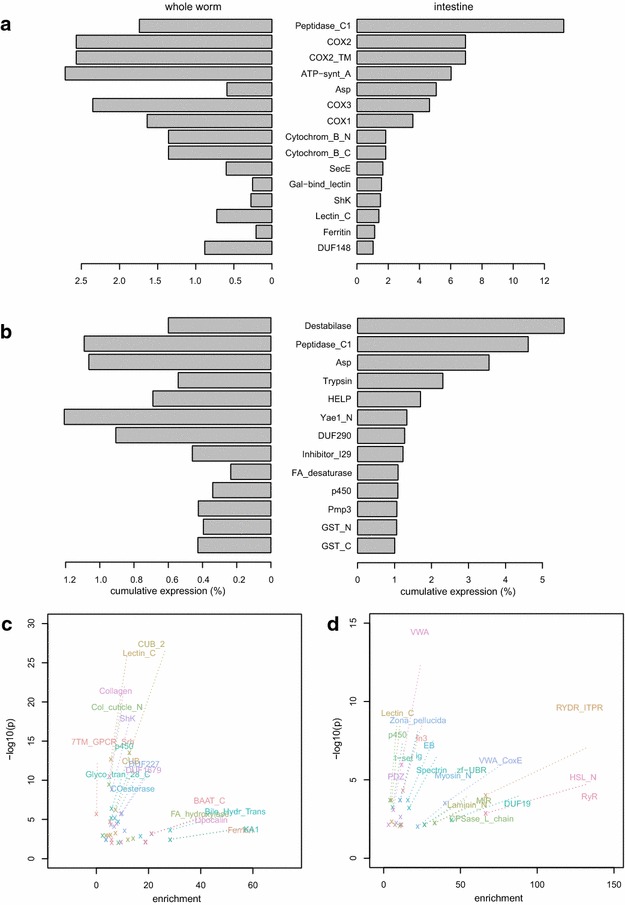
Divergence at the gene family level. **a** Cumulative expression of gene families in *C. elegans*. Cumulative expression was defined as the sum of FPKM values for a given gene family divided by the sum of all FPKM values and only families with a cumulative expression above 1 % in the intestinal transcriptome that simultaneously have a higher cumulative expression as opposed to the whole worm transcriptome are shown. **b** Cumulative expression of gene families in *P. pacificus*. **c** Gene family enrichment plot for the intestine-enriched *C. elegans* genes. **d** Gene family enrichment plot for the intestine-enriched *P. pacificus* genes

Despite the presence of some conserved patterns, the overwhelming trend in our comparative analysis seems to be a strong divergence of transcriptomic profiles at the single-gene level. However, even in the presence of functional divergence at a single-gene level, conserved functions may be performed by other members of a given gene family. We first defined gene families based on the presence of a given protein domain (PFAM) and compared the cumulative expression (sum of FPKM values for a given gene family divided by the sum of all FPKM values) between the two species (Fig. 3a, b). While cysteine proteases (peptidase_C1, PF00112) make the most abundant transcript accounting for up to 13 % of all intestinal transcripts in *C. elegans*, this family also represents the second most abundant gene family in the intestinal transcriptome of *P. pacificus*. Similarly Aspartate protease (PF00026) show a comparably high expression level in the intestinal transcriptomes of both species. However, the majority of gene families that account for at least 1 % of the intestinal transcriptomes seems to be species-specific (Fig. 3a, b). While the cumulative expression of gene families as displayed in Fig. 3a, b is a rather descriptive measure that is influenced by the differences in gene family size and is not normalized against the whole worm transcriptome, we further investigated the conservation of intestine-specific expression at a gene family level by testing whether the same families are enriched in intestine-specific genes in *C. elegans* as well as in *P. pacificus*, i.e. genes that are significantly higher expressed in the intestinal transcriptome of the respective species. In total, we detected 45 and 28 gene families that are enriched in the intestinal transcriptomes of *C. elegans* and *P. pacificus*, respectively (Fig. 3c, d). Yet, there are only five protein domains that are significantly enriched ($P<0.01$) in the intestinal transcriptomes of both species: CUB domains (PF00431), Cytochromes p450 (PF00067), C-type Lectins (PF00059), Collagens (PF01391), and VWA domains (PF00092). In *C. elegans* CUB domain containing proteins are significantly enriched in the Gene ontology biological process proteolysis (GO:0006508, $P < 10^{-5}$), p450 proteins are enriched in oxidation reduction processes (GO:0055114, $P < 10^{-89}$ ), C-type Lectins are enriched in positive regulation of growth (GO:0045927, $P<0.05$), collagens are enriched for body morphogenesis (GO:0010171, $P < 10^{-30}$), only VWA domain containing protein do not show any significant enrichment in any biological process in *C. elegans*. In summary, similar to the analysis at the single-gene level, the low number of common gene families and the fact that the most highly significantly enriched gene families do not match across the two species demonstrate a substantial degree of transcriptomic divergence even at the gene family level (Fig. 3c, d).

### Intestinal luminal pH is maintained despite transcriptomic divergence

**Fig. 4:**
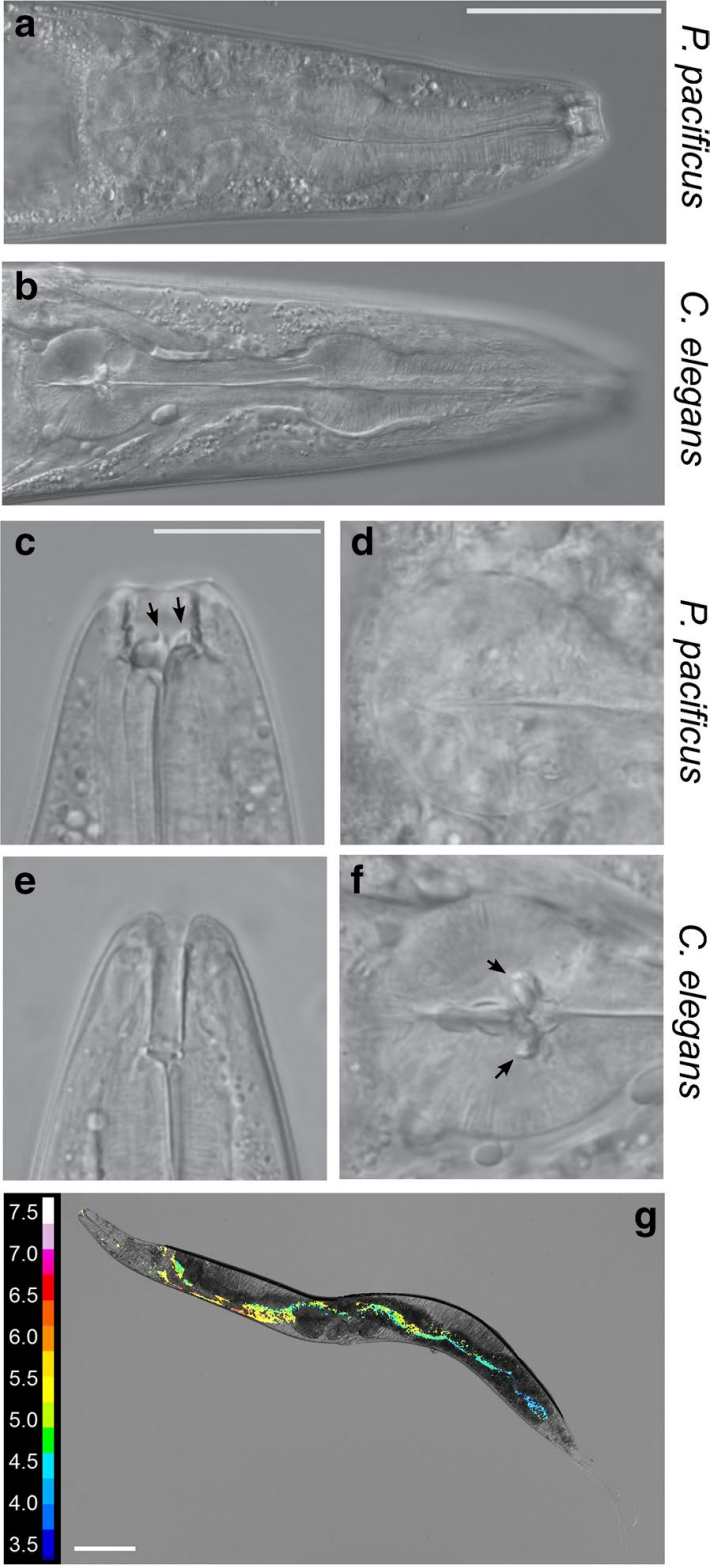
Morphological differences in feeding structures. **a** Whole *P. pacificus* pharyngeal structure including specialised predatory feeding adaptations (*scale bar* = 50 $\upmu$m). **b** Whole *C. elegans* pharynx **c** Higher magnification image of *P. pacificus* specialised teeth-like feeding adaptations facilitating predatory feeding (*scale bar* = 20 $\upmu$m). **d** Terminal bulb of *P. pacificus* pharynx with no grinder present **e** *C. elegans* tube like mouth cavity and **f** grinder in the terminal bulb of *C. elegans* to aid with bacterial lysis. **g** Transformed false color pH heat map image for *P. pacificus*. *Scale bar* = 100 $\upmu$m

As mentioned previously, in addition to the presence of teeth-like structures in *P. pacificus* (Fig. 4a–f), an important anatomical difference between *C. elegans* and *P. pacificus* is that *C. elegans* worms have a specialised grinder structure located in the posterior bulb of the pharynx (Fig. 4a–f) which is involved in the physical lysis of bacterial food. While the grinder is a typical structure of nematodes of the Rhabditidae family, no grinder exists in nematodes of the Diplogastridae family, to which *P. pacificus* belongs. Consequently, living bacteria can be observed in the gut of *P. pacificus* [32]. Thus, how *P. pacificus* is able to break open and extract nutrients from the ingested bacteria remains unknown. However, with such striking differences in transcriptome profile between these nematode species we speculated that the internal environment may differ along the length of the nematode intestine. We therefore focused on the intestinal pH in *P. pacificus* as it provided a simple comparison between species as it is possible to observe real-time dynamic changes in pH using extended dynamic range pH-sensitive nanosensors [20]. In order to measure potential differences in intestinal pH between species, the pH-sensitive nanosensors were injected into the lumen of the *P. pacificus* intestine and the ratio of fluorescence from the nanosensors was used to quantify the pH throughout the intestine (Fig. 4g). Despite the extreme transcriptomic variation between the species, we could not detect any large difference in pH between *P. pacificus* and *C. elegans* along the length of the intestine. In both species the initial anterior pH was close to pH 6 before decreasing as low as pH 3.5 toward the posterior of the animal. Thus, the pH gradient along the intestinal tract remains conserved between species. Therefore diverse bacterial lysis and nutrient extraction methods between species likely function under similar intestinal pH concentrations.

### Transcriptomic divergence is reflected in differential response to pathogens

While it remains unclear, how exactly *P. pacificus* gains nutrition from its bacterial food, the absence of the grinder indicates a global shift in feeding mode with important ecologically and evolutionary consequences. As the physical lysis of bacteria in the posterior part of the pharynx suggests an immediate release of all bacterial toxins, it may be speculated that the absence of the grinder in *P. pacificus* avoids the release of high concentrations of bacterial toxins into the anterior part of the intestine. As a consequence, *P. pacificus* worms should be less susceptible at least to some bacterial pathogens as the effect of bacterial toxins is much more pronounced in the intestine in *C. elegans* and similarly that its intestine must harbor defense mechanisms. The greater resistance of *P. pacificus* to various bacterial pathogens was shown by a previous study [7], investigating survival and transcriptomic profiles of *C. elegans* and *P. pacificus* nematodes in response to exposure to four different pathogens: *Bacillus thuringiensis*, *Staphylococcus aureus*, *Serratia marcescens*, *Xenorhabdus nematophila*. Using our transcriptomic data, we can now test whether indeed in *C. elegans* more genes that are differentially expressed upon pathogen exposure are also enriched in the intestine (Table 2).

**Table 2:** Intestine-enriched genes in both species were tested for overlap with genes differentially expressed upon pathogen exposure

Nematode	Pathogen/response	Common genes	Enrichment	*P*-value
*C. elegans*	*B. thuringiensis*/ down	29	5.4	<10^−12^
*C. elegans*	*B. thuringiensis*/ up	209	1.1	n.s.
*C. elegans*	*S. aureus*/ down	18	7.5	<10^−10^
*C. elegans*	*S. aureus*/ up	43	7.0	<10^−23^
*C. elegans*	*S. marcescens*/ down	114	0.7	<10^−6^
*C. elegans*	*S. marcescens*/ up	198	3.9	<10^−64^
*C. elegans*	*X. nematophila*/ down	403	1.5	<10^−6^
*C. elegans*	*X. nematophila*/ up	54	2.2	<10^−6^
*P. pacificus*	*B. thuringiensis*/ down	5	5.5	0.02
*P. pacificus*	*B. thuringiensis*/ up	6	2.6	n.s.
*P. pacificus*	*S. aureus*/ down	3	1.4	n.s.
*P. pacificus*	*S. aureus*/ up	8	3.0	0.03
*P. pacificus*	*S. marcescens*/ down	19	1.3	n.s.
*P. pacificus*	*S. marcescens*/ up	4	1.4	n.s.
*P. pacificus*	*X. nematophila*/ down	81	1.3	n.s.
*P. pacificus*	*X. nematophila*/ up	6	0.5	n.s.

Strikingly, while none of the comparisons for *P. pacificus* showed a highly significant enrichment of intestine-enriched genes among genes differentially expressed upon pathogen exposure (*P* < 0.01), six out of eight comparisons showed highly significant associations between pathogen response and intestine-enriched genes. The only two exceptions consisted in comparisons with the two pathogens that killed *C. elegans* nematodes most efficiently, thus these transcriptomes are likely dominated by secondary effects such as pathogenesis related necrosis of host-tissues. In summary, our analysis clearly shows that intestine-enriched genes are associated with pathogen response in *C. elegans* but not in *P. pacificus*, which indicates that morphological differences in their feeding structures are paralleled by differences in pathogen susceptibilty.

## Discussion

In this study, we have investigated the intestinal transcriptomes of the nematodes *C. elegans* and *P. pacificus*. Our approach used RNA obtained from intact animals as control to screen for genes that are preferentially expressed in the intestinal sample. However, failure to detect the expression of a gene in the intestine, does not imply that the gene does not play a functionally important role in the intestine. Thus, many genes may be missed just because their overall expression level is not significantly different between the intestine and the complete animal or alternatively because of low statistical power (low expression, only one replicate). Our analysis showed that at least for a subset of genes, lack of conserved intestine-specific expression is due to broad expression in the other lineage (Fig. 2b, c). Thus, the identified gene sets provides just a footprint of the strongest intestine-specific expression and can be used for a first comparative analysis but they do not represent a complete catalogue of intestine-enriched genes. The fact, that previous studies have used quite distinct approaches to obtain tissue-specific transcriptomes in *C. elegans* [23–26], indicates that these kind of studies are inherently difficult and upscaling to more tissues and replicates only becomes feasible if sample and library preparation protocols further improve.

Despite the fact, that the identified gene sets are far from being complete, our first analysis shows substantial divergence between the genes with strongest intestine-enriched expression in both species. More precisely, only 15 genes with one-to-one orthologs were identified as intestine-enriched in both species (Fig. 2a), indicating that the largest fraction of intestine-enriched genes derived from lineage-specific events. Similarly, the strongest overrepresentations of gene families among intestine-enriched genes also seems to be lineage-specific (Fig. 3c, d), a pattern that recapitulates findings from studying the developmental transcriptomes of *C. elegans* and *P. pacificus* [3, 19]. Nevertheless, our analysis reveals highly conserved expression of certain Hedgehog signaling genes, which together with findings from other animal phyla [29, 30] points to an ancient and highly conserved function of Hedgehog signaling across all metazoans.

Given, that *C. elegans* and *P. pacificus* show strong morphological differences in their pharyngeal anatomy, i.e. the absence of the grinder in the *P. pacificus* lineage, which has been hypothesized to play a role in the susceptability to various pathogens [3], we compared the sets of intestine-enriched genes to genes that are differentially expressed upon pathogen exposure. Unexpectedly, we see a very striking trend as for *C. elegans*, there are highly significant overlaps between gene sets, while for *P. pacificus* there is not a single highly significant overlap ($P<0.01$). It has been shown that *P. pacificus* is more resistant to several pathogens compared to *C. elegans* [3] and it can be speculated that this is at least partially due to the sudden release of bacterial toxin upon physical lysis in *C. elegans*. Our data is largely consistent with this hypothesis and supports the idea of the lack of grinder as a mechanistic explanation for the increased resistance. However, the correlations that we see do not represent an experimental proof.

It has to be mentioned that the grinder serves to disrupt bacterial cell walls and to gain nutritients but it also serves as physical barrier to kill pathogenic bacteria and to prevent them from establishing intestinal infections [33]. However, at least for intestinal pathogens such as *S. marcescens*, it has been shown that intestinal infections are facilitated by first interferring with the function of the grinder [34]. More precisely, while normally, intact GFP labeled *E. coli* OP50 bacteria could not be observed in the gut of *C. elegans* worms, short exposure to a strain of *S. marcescens* enables fluorescent OP50 to pass the grinder [34]. In addition, pathogenicity mechanisms can be very different even within a single pathogen. *Pseudomonas aeruginosa* for example has two different modes of killing *C. elegans*. A slow killing mode that functions via an infection-like proccess in the intestine and a fast toxin-based killing mode [35]. Our interpretation of the lack of the grinder as a means to avoid high concentrations of bacterial toxins might therefore be better suited to explain the increased resistance to toxin-based pathogenicity mechanisms.

Taken together, The susceptibility to pathogens might rather be a question of being able to maintain the microbiome composition at correct concentrations throughout the intestine and both species might have developed different control mechanisms given their anatomy. Thus, any perturbation may cause a suboptimal state leading to an increased susceptibility. This is shown by the fact that grinder-less mutants or mutations affecting intestinal peristalsis show increased susceptibility at least to certain pathogens [8]. In adition, to better understand the differences in pathogen suceptibility between both nematodes, we have to know how long bacteria stay resident in both species. Although comparisons of reported pumping rates and defectation cycles suggest differences between the species [8, 36, 37], a comprehensive analysis of bacterial residence times in both species is still lacking.

## Conclusions

Our study reveals first insights into the evolution of feeding systems and the associated changes in intestinal gene expression and it provides support for the idea that anatomical differences might have facilitated nematodes of the *P. pacificus* lineage to colonize new habitats such as decaying beetle carcasses. These findings deepen our understanding about how morphological and genomic diversity is created during the course of evolution.

## Additional file

10.1186/s13104-016-1886-9 Gene lists of intestine-enriched genes.
